# Web-based dynamic nomogram for forecasting overall survival and cancer-specific survival among individuals with lymph node-negative pancreatic cancer: based upon the SEER database

**DOI:** 10.1016/j.clinsp.2026.101100

**Published:** 2026-09-07

**Authors:** Zheyuan Wang, Lu Zhang, Zengyou Li, Huihan Zhang

**Affiliations:** a Department of General Surgery, The Second Hospital of Lanzhou University, Lanzhou, Gansu, China; b Department of Ultrasound Diagnosis, Gansu Provincial Maternity and Child-care Hospital, Lanzhou, Gansu, China; c Department of Pathology, The Second Hospital of Lanzhou University, Lanzhou, Gansu, China

**Keywords:** Competing risk model, Predictive nomogram, Lymph node-negative pancreatic cancer, SEER, Cancer-specific death

## Abstract

**Background:**

This study aimed to develop and validate prognostic models for Lymph Node (LN)-negative pancreatic cancer patients.

**Methods:**

Data were extracted from the SEER database (2004‒2015). The included participants were randomly divided into training (70%) and validation (30%) sets. Independent prognostic factors for Overall Survival (OS) and Cancer-Specific Survival (CSS) were identified using Cox and Fine-Gray models to construct predictive nomograms for 1-, 3-, and 5-year outcomes.

**Results:**

Among 5970 included patients, 4270 deaths occurred. The nomogram for OS included 11 variables and the nomogram for CSS included 10. For OS, the C-indices in the training and validation cohorts were 0.740 (95% CI 0.722–0.758) and 0.740 (95% CI 0.712–0.768), respectively. Similarly, the C-indices for CSS were 0.737 (95% CI 0.719–0.756) and 0.736 (95% CI 0.708–0.764), respectively. The AUCs for 1-, 3-, and 5-year OS were 0.794 (95% CI 0.770–0.818), 0.819 (95% CI 0.800–0.839), and 0.836 (95% CI: 0.816–0.855). Meanwhile, the AUCs for 1-, 3-, and 5-year CSS were 0.796 (95% CI: 0.781–0.812), 0.829 (95% CI: 0.816–0.841), and 0.850 (95% CI: 0.838–0.862), indicating strong predictive performance. Calibration curves confirmed good accuracy. Competing risk analysis showed conventional methods overestimated CSS, supporting the accuracy of the Fine-Gray model.

**Conclusion:**

We developed and internally validated two clinically practical nomograms for OS and CSS for patients with LN-negative pancreatic cancer. These models show favorable discrimination and calibration, enabling clinical risk stratification and prognosis assessment. Future external validation using independent cohorts is needed to confirm the generalizability of these models.

## Highlights

• Developed nomograms predicting OS and CSS in LN-negative pancreatic cancer.
• The Fine‑Gray model improved accuracy of cancer-specific survival estimates.
• Nomograms integrated key clinicopathological factors with high predictive accuracy.
• An online calculator enables individualized prognosis assessment in clinic.

## Introduction

Pancreatic cancer is the seventh predominant cause of cancer-attributable mortality globally, with its incidence demonstrating an upward trend. Notably, the incidence in developed countries is at least 5-fold higher than in developing nations.^1^ In the USA, pancreatic cancer is the third predominant cause of cancer-attributable mortality in both sexes, representing approximately 47,906 mortalities in 2021. The relative survival rate within five years is solely 13%.^2^ Adenocarcinoma represents the predominant histologic subtype of pancreatic cancer, comprising roughly 90% of cases. Other types include indolent neuroendocrine tumors and rare pancreatic exocrine carcinomas.^3^ High‑risk factors for the progression of pancreatic cancer encompass smoking, obesity, alcohol consumption, diabetes, and chronic pancreatitis.^4^^,^^5^ Currently, the primary therapeutic approaches for pancreatic cancer include surgery and chemotherapy. However, most patients present with metastatic disease upon diagnosis. Solely 15%–20% are eligible for surgical resection. Even among patients undergoing resection, 75% experience recurrence within twenty-four months.^6^ Factors influencing the prognosis of pancreatic cancer include unresectable tumor, perineural and vascular invasion, lymph node metastasis, postoperative recurrence, and genetic factors. Notably, lymph node metastasis serves as a critical indicator for advanced-stage cancer and tumor recurrence.^7^^,^^8^ Though some studies suggest that patients with lymph node-negative pancreatic cancer may experience longer postoperative survival,^7^ substantial heterogeneity in prognosis is observed within this population. Some cases still face elevated risks of early recurrence and death after surgery. Consequently, based upon the relatively favorable pathological characteristic of lymph node negativity, it is necessary to develop a more precise prognostic evaluation system to identify high‑risk populations and to guide individualized adjuvant treatments and follow‑up strategies.

In this context, the traditional TNM (Tumor size, Nodal status, Metastasis) staging system relies on the anatomic extent of malignancy. It cannot fully incorporate other clinicopathological variables that have been demonstrated to possess significant prognostic value (like age, sex, and therapeutic response). This limitation reduces its utility for refined risk stratification and individualized prognostic prediction. As a visualization tool for multivariable statistical prediction models, the nomogram integrates multiple independent prognostic factors and assigns different weights to each predictor based upon their contributions to clinical endpoints. It ultimately presents the probability of specific events (like Overall Survival [OS] or relapse-free survival) for individual patients in an intuitive graphical format.^9^^,^^10^ Recently, substantial evidence has demonstrated the clinical utility of nomogram-based models in prognostic prediction for various malignancies, encompassing lung, breast, and colorectal cancers.^11^, ^12^, ^13^ However, reliable prognostic tools remain lacking for the specific cohort of individuals with lymph node-negative pancreatic cancer. Therefore, this research developed, and validated nomogram-based prediction models specifically tailored for these populations based upon the Surveillance, Epidemiology, and End Results (SEER) database from the National Cancer Institute (NCI) of the USA, thereby providing a more intuitive and individualized risk assessment tool for clinical practice.

## Materials and methods

### Data source and data extraction

The data on patients in this research were attained from the SEER database of the NCI of the USA (https://seer.cancer.gov/seerstat/). As a cancer registry program in the USA, SEER covered approximately 28% of the US population and included data from 18 cancer registries. It provided demographic information, clinicopathological characteristics, and survival status of patients. The clinicopathological information utilized in this research was publicly available and de‑identified. Consequently, ethical approval and informed consent were not necessary for this research. Our methodology complied with the requirements of the SEER database.

This study identified 123,104 cases diagnosed with pancreatic cancer from 2004 to 2015. We collected information on age, sex, race, marital status, year of diagnosis, tumor size, primary tumor site, histologic type, pathological grade, tumor stage, surgery, radiotherapy, chemotherapy, vital status, and survival time. Exclusion criteria were outlined below: i) Patients with lymph node-positive pancreatic cancer; ii) Duration of follow‑up was less than one month; iii) Individuals with uncertain cause of death; iv) Individuals with missing information on tumor stage, type of surgery, or survival time.

The covariates in this study included sex, age, race, marital status, tumor size, primary tumor site, histologic type, pathological grade, TNM staging system, surgery, chemotherapy, and radiotherapy. All included cases had adequate follow‑up. The primary endpoints were OS and Cancer Specific Death (CSD). OS was defined as the time from the date of diagnosis to death from any cause. CSD was defined as the interval from the date of diagnosis of pancreatic cancer to death attributed to pancreatic cancer.

To minimize the risk of overfitting, we performed multivariable analyses in accordance with the Events-Per-Variable (EPV) principle, requiring at least 10 outcome events per predictor variable included in the model.

### Statistical analysis

All patients were randomly classified into a training cohort (70%) and a validation cohort (30%). A table of baseline characteristics was constructed for the training and validation cohorts. Categorical variables were expressed as counts and percentages. Inter-group comparisons were implemented utilizing the Chi-Square test. Normally distributed continuous variables were expressed as mean ± standard deviation (x̄±s). Inter-group comparisons were implemented utilizing the independent-samples *t*-test. Non-normally distributed continuous variables were expressed as Median (Interquartile Range) [M(IQR)]. Inter-group comparisons were implemented utilizing the Mann-Whitney *U*-test.

Cox proportional hazards models and Fine-Gray models were used to identify independent prognostic risk indicators for OS and Cancer-Specific Survival (CSS) among individuals with lymph node-negative pancreatic cancer. Nomograms for OS and CSS were then constructed based upon the identified independent risk indicators. The Concordance index (C-index), the Area Under the ROC Curve (AUC), and calibration plots were employed to estimate the accuracy and discriminative capability of the models. The C-indices ranged from 0.5 to 1. A prediction model with a C-index greater than 0.7 was defined as favorable. Calibration plots were used to evaluate the agreement between predicted and observed outcomes, with calibration for the Fine-Gray model assessed using Cumulative Incidence Functions (CIF). Decision Curve Analysis (DCA) was employed to evaluate the clinical utility of the constructed prediction model. Furthermore, DCA was used to compare the model with TNM staging system to evaluate the incremental value of the model.

Finally, this research developed an online dynamic prediction tool based upon the constructed nomograms. By implementing the DynNom package in R, this tool generated an interactive visualization interface that provided real-time prediction results when individual clinical characteristics were input. The application of the dynamic model was deployed on the shinyapps.io platform to enable online access and instantaneous prediction.

In accordance with the lymph node staging criteria (≥12 lymph nodes) recommended by the 8th Edition guidelines of the American Joint Committee on Cancer (AJCC), we performed stratified sensitivity analyses. Patients were stratified into two groups based on the number of examined lymph nodes: ≥ 12 and < 12. All analyses were restricted to lymph node-negative patients to further validate the stability of the model. In each subgroup, the AUC was used to evaluate the discriminatory performance of the prognostic model.

Furthermore, sensitivity analyses restricted to M0 patients were performed to evaluate the robustness of our prognostic models and ensure its clinical relevance for non-metastatic patients.

All statistical analyses were implemented utilizing R version 4.4.2; p < 0.05 signified statistical significance. This prognostic study followed the STARD guidelines.

## Results

### Clinical characteristics of patients

In total, 5970 cases were included. These were randomly classified into a training cohort (n = 4179, 70%) and a validation cohort (n = 1791, 30%). Among these patients, 50.1% were male and 49.9% were female. Regarding race, 80.3% were White. In terms of age distribution, 13.7% of patients were < 50-years, 52.8% were between 50- and 70-years, and 33.5% were > 70-years. Locations of the tumor included the pancreatic head (59.5%), body (10.6%), and tail (17.5%). The proportion of cases with a histologic type of adenocarcinoma was 51.1%. A total of 53.0% of patients received chemotherapy, while the majority did not receive radiotherapy (71.9%). Pancreaticoduodenectomy was performed in 51.7% of patients, and total pancreatectomy was conducted in 9.30% of patients (Table 1). No marked differences were observed between the training and validation cohorts for any variable (all p > 0.05).

**Table 1:** Baseline clinical characteristics of individuals with lymph node-negative pancreatic cancer.

	**Overall** **(n = 5970)**	**Train** **(n = 4179)**	**Test** **(n = 1791)**	**p**
**Sex**				0.857
Female	2981 (49.9%)	2083 (49.8%)	898 (50.1%)	
Male	2989 (50.1%)	2096 (50.2%)	893 (49.9%)	
**Age**				0.967
< 50	820 (13.7%)	577 (13.8%)	243 (13.6%)	
50‒70	3153 (52.8%)	2204 (52.7%)	949 (53.0%)	
70+	1997 (33.5%)	1398 (33.5%)	599 (33.4%)	
**Race**				0.547
Black	592 (9.92%)	416 (9.95%)	176 (9.83%)	
Other	584 (9.78%)	420 (10.1%)	164 (9.16%)	
White	4794 (80.3%)	3343 (80.0%)	1451 (81.0%)	
**Marital status**				0.892
Married	3795 (63.6%)	2660 (63.7%)	1135 (63.4%)	
Other	1318 (22.1%)	925 (22.1%)	393 (21.9%)	
Unmarried	857 (14.4%)	594 (14.2%)	263 (14.7%)	
**Primary tumor site**				0.490
Head of pancreas	3555 (59.5%)	2510 (60.1%)	1045 (58.3%)	
Body of pancreas	630 (10.6%)	442 (10.6%)	188 (10.5%)	
Tail of pancreas	1045 (17.5%)	712 (17.0%)	333 (18.6%)	
Other	740 (12.4%)	515 (12.3%)	225 (12.6%)	
**Hist**				0.470
Adenocarcinoma	3051 (51.1%)	2149 (51.4%)	902 (50.4%)	
Other	2919 (48.9%)	2030 (48.6%)	889 (49.6%)	
**Grade**				0.164
Grade I	1280 (21.4%)	871 (20.8%)	409 (22.8%)	
Grade II	2040 (34.2%)	1418 (33.9%)	622 (34.7%)	
Grade III‒IV	1276 (21.4%)	917 (21.9%)	359 (20.0%)	
Unknown	1374 (23.0%)	973 (23.3%)	401 (22.4%)	
**AJCC T**				0.956
T1	910 (15.2%)	635 (15.2%)	275 (15.4%)	
T2	1423 (23.8%)	989 (23.7%)	434 (24.2%)	
T3	3057 (51.2%)	2149 (51.4%)	908 (50.7%)	
T4	580 (9.72%)	406 (9.72%)	174 (9.72%)	
**AJCC M**				0.415
M0	5441 (91.1%)	3800 (90.9%)	1641 (91.6%)	
M1	529 (8.86%)	379 (9.07%)	150 (8.38%)	
**Surgery**				0.263
No	936 (15.7%)	659 (15.8%)	277 (15.5%)	
Other	1393 (23.3%)	952 (22.8%)	441 (24.6%)	
Pancreatoduodenectomy	3086 (51.7%)	2164 (51.8%)	922 (51.5%)	
Total pancreatectomy	555 (9.30%)	404 (9.67%)	151 (8.43%)	
**Size**				0.087
0‒30	3028 (50.7%)	2114 (50.6%)	914 (51.0%)	
31‒50	1966 (32.9%)	1397 (33.4%)	569 (31.8%)	
51‒70	552 (9.25%)	363 (8.69%)	189 (10.6%)	
70+	424 (7.10%)	305 (7.30%)	119 (6.64%)	
**Radiotherapy**				0.343
None	4292 (71.9%)	3020 (72.3%)	1272 (71.0%)	
Yes	1678 (28.1%)	1159 (27.7%)	519 (29.0%)	
**Chemotherapy**				0.387
Unknown	2804 (47.0%)	1947 (46.6%)	857 (47.9%)	
Yes	3166 (53.0%)	2232 (53.4%)	934 (52.1%)	
Time	51.4 (49.5)	51.1 (49.4)	51.9 (49.9)	0.586
**OS**				0.511
Alive	1700 (28.5%)	1201 (28.7%)	499 (27.9%)	
Dead	4270 (71.5%)	2978 (71.3%)	1292 (72.1%)	
**CSS**				0.627
Alive	1700 (28.5%)	1201 (28.7%)	499 (27.9%)	
Death for pancreatic cancer	3749 (62.8%)	2608 (62.4%)	1141 (63.7%)	
Death for others	521 (8.73%)	370 (8.85%)	151 (8.43%)	

### Selection of prognostic indicators for OS

First, a univariate Cox regression model was developed. The results exhibited that sex, age, marital status, primary tumor site, histologic type, pathological grade, AJCC TNM stage, surgery, tumor size, radiotherapy, and chemotherapy all demonstrated marked distinctions in the univariate analysis (Table 2). Subsequently, variables with statistical significance in the univariate Cox regression analysis were incorporated into a multivariate Cox regression model to identify independent risk indicators affecting OS. The results indicated that sex, age, marital status, primary tumor site, pathological grade, AJCC T stage, AJCC M stage, histologic type, surgery, tumor size, and chemotherapy were independent risk indicators (Table 2).

**Table 2:** Univariate and multivariate analyses of OS in lymph node-negative pancreatic cancer.

**Factors**	**Define**	**Univariate**		**Multivariate**	
		**HR (95% CI)**	***z*(p)**	**HR (95% CI)**	***z*(p)**
Sex	Female	Ref.		Ref.	
	Male	1.13 (1.05‒1.22)	3.38 (<0.001)	1.09 (1.01‒1.17)	2.20 (0.029)
Age	< 50	Ref.		Ref.	
	50‒70	2.13 (1.86‒2.45)	10.91 (<0.001)	1.62 (1.40‒1.86)	6.69 (<0.001)
	70+	3.38 (2.94‒3.88)	17.14 (<0.001)	2.53 (2.18‒2.92)	12.46 (<0.001)
Marital status	Married	Ref.		Ref.	
	Unmarried	0.85 (0.76‒0.95)	−2.84 (0.004)	1.079 (0.95‒1.20)	1.14 (0.300)
	Other	1.31 (1.20‒1.43)	6.20 (<0.001)	1.17 (1.07‒1.28)	3.42 (<0.001)
Race	Black	ref.			
	White	1.11 (0.98‒1.26)	1.66 (0.100)		
	Other	0.92 (0.78‒1.09)	−0.99 (0.300)		
Primary tumor site	Head of pancreas	Ref.		Ref.	
	Body of pancreas	0.65 (0.57‒0.73)	−6.81 (<0.001)	0.94 (0.82‒1.08)	−0.87 (0.400)
	Tail of pancreas	0.46 (0.41‒0.52)	−13.43 (<0.001)	0.83 (0.72‒0.96)	−2.60 (0.009)
	Other	0.78 (0.70‒0.88)	−4.26 (<0.001)	1.06 (0.94‒1.19)	0.89 (0.400)
Grade	Grade I	Ref.		Ref.	
	Grade II	2.84 (2.52‒3.21)	16.89 (<0.001)	1.99 (1.75‒2.26)	10.58(<0.001)
	Grade III‒IV	4.11 (3.62‒4.67)	21.76 (<0.001)	2.65 (2.32‒3.04)	14.07 (<0.001)
	Unknown	3.23 (2.84‒3.67)	17.97(<0.001)	1.57 (1.37‒1.81)	6.31 (<0.001)
AJCC_T	T1	Ref.		Ref.	
	T2	1.78 (1.53‒2.06)	7.61 (<0.001)	1.49 (1.27‒1.74)	4.98 (<0.001)
	T3	3.43 (3.01‒3.92)	18.30 (<0.001)	2.06 (1.78‒2.38)	9.75 (<0.001)
	T4	6.31 (5.38‒7.42)	22.46 (<0.001)	2.45 (2.04‒2.93)	9.67 (<0.001)
AJCC_M	M0	Ref.		Ref.	
	M1	2.62 (2.34‒2.94)	16.65 (<0.001)	1.85 (1.63‒2.10)	9.64 (<0.001)
Surgery	No Surgery	Ref.		Ref.	
	Pancreatoduodenectomy	0.27 (0.24‒0.29)	−27.06 (<0.001)	0.33 (0.30‒0.38)	−17.68 (<0.001)
	Total pancreatectomy	0.24 (0.21‒0.28)	−19.35 (<0.001)	0.33 (0.28‒0.39)	−13.31 (<0.001)
	Other	0.14 (0.13‒0.16)	−30.87 (<0.001)	0.29 (0.25‒0.34)	−14.79 (<0.001)
Radiotherapy	None	Ref.		Ref.	
	Yes	1.46 (1.35‒1.57)	9.59 (<0.001)	1.07 (0.98‒1.17)	1.45 (0.150)
Chemotherapy	Unknown	Ref.		Ref.	
	Yes	1.78 (1.65‒1.92)	15.13 (<0.001)	0.90 (0.82‒0.98)	−2.37 (0.018)
Size	0‒30	Ref.		Ref.	
	31‒50	1.63 (1.51‒1.76)	12.16 (<0.001)	1.23 (1.13‒1.34)	4.84 (<0.001)
	51‒70	1.35 (1.19‒1.54)	4.57 (<0.001)	1.11 (0.97‒1.27)	1.49 (0.140)
	70+	0.92 (0.79‒1.07)	−1.11 (0.300)	1.03 (0.88‒1.21)	0.33 (0.700)
Hist	Adenocarcinoma	Ref.		Ref.	
	Other	0.43 (0.40‒0.47)	−20.87 (<0.001)	0.74 (0.68‒0.80)	−7.34 (<0.001)

### Nomogram for OS and its validation

**Fig. 1:**
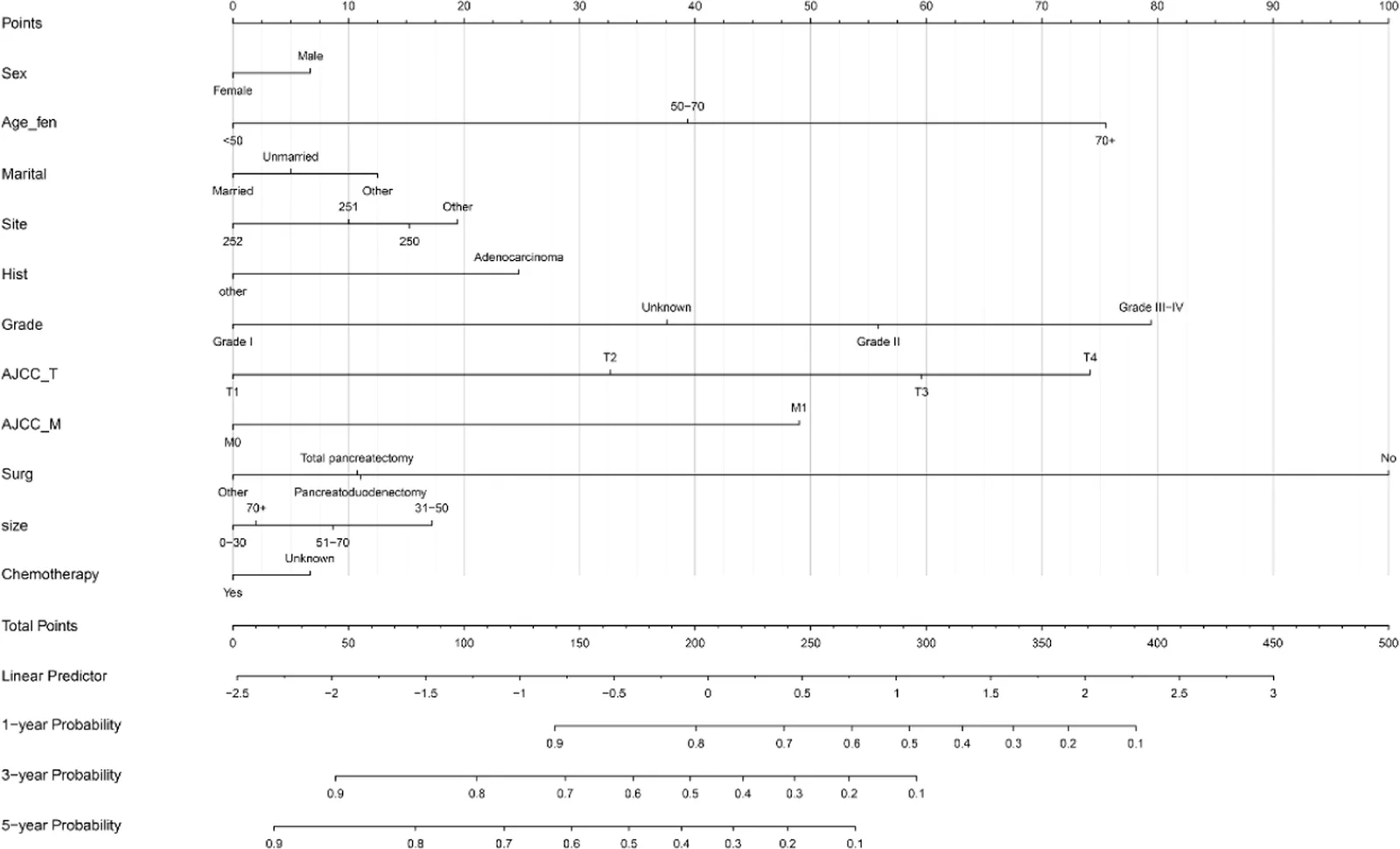
Nomogram for forecasting one-, three-, and five-year OS.

Based upon the independent risk indicators identified above, a nomogram was developed to forecast OS (Fig. 1). The nomogram quantitatively visualized the contribution of each factor to OS. Surgery was identified as the most important predictor, followed by the pathological grade of pancreatic cancer. Poorly differentiated and undifferentiated tumors were linked to markedly increased risks of mortality. AJCC T and M stage, as well as age > 70-years, were also important risk indicators. In addition, female patients, individuals with tumors situated in the pancreatic tail, those with a tumor diameter < 30 mm, and those who received chemotherapy had relatively lower mortality.

**Fig. 2:**
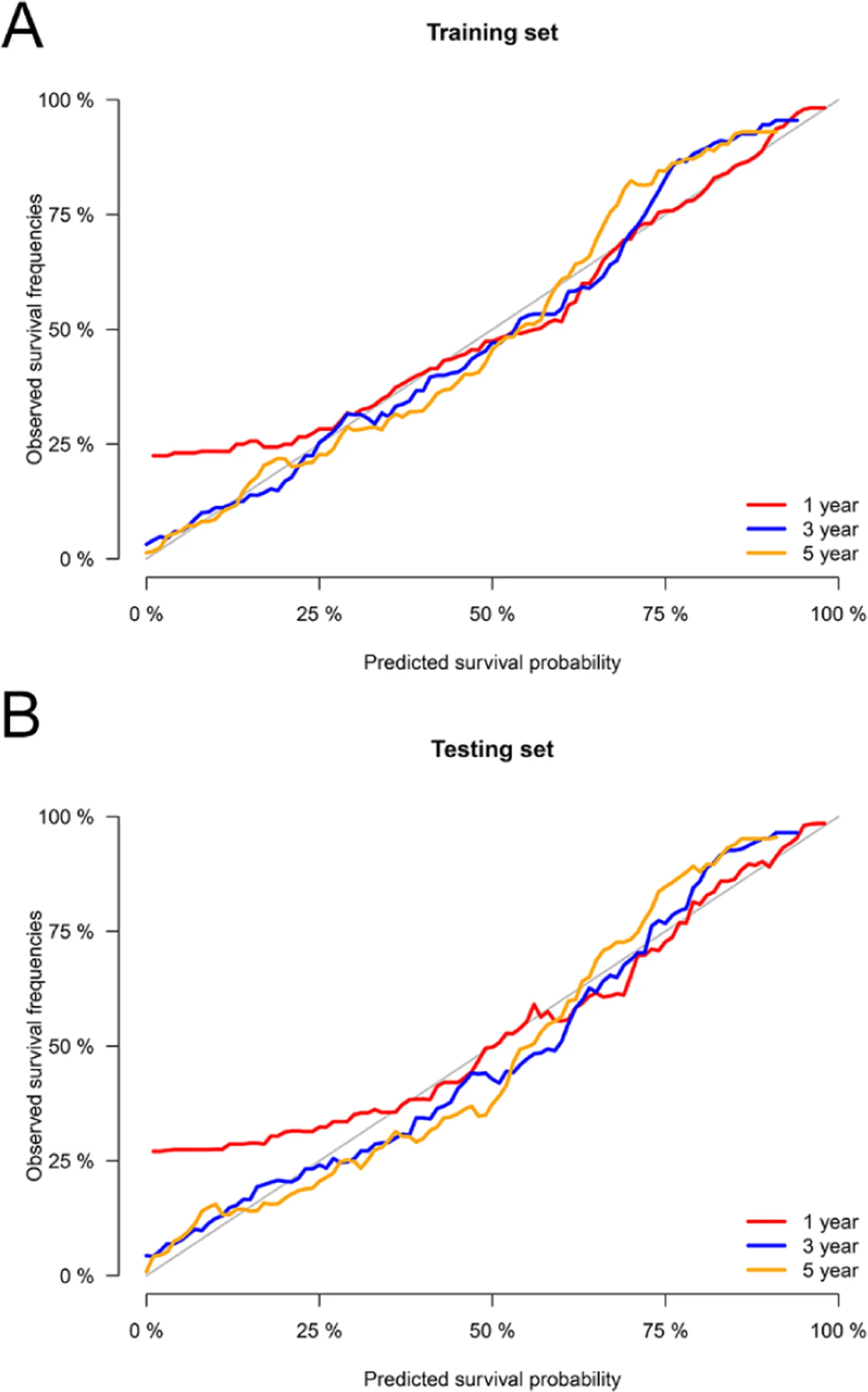
Calibration curves for the training cohort (A), and the validation cohort (B).

**Fig. 3:**
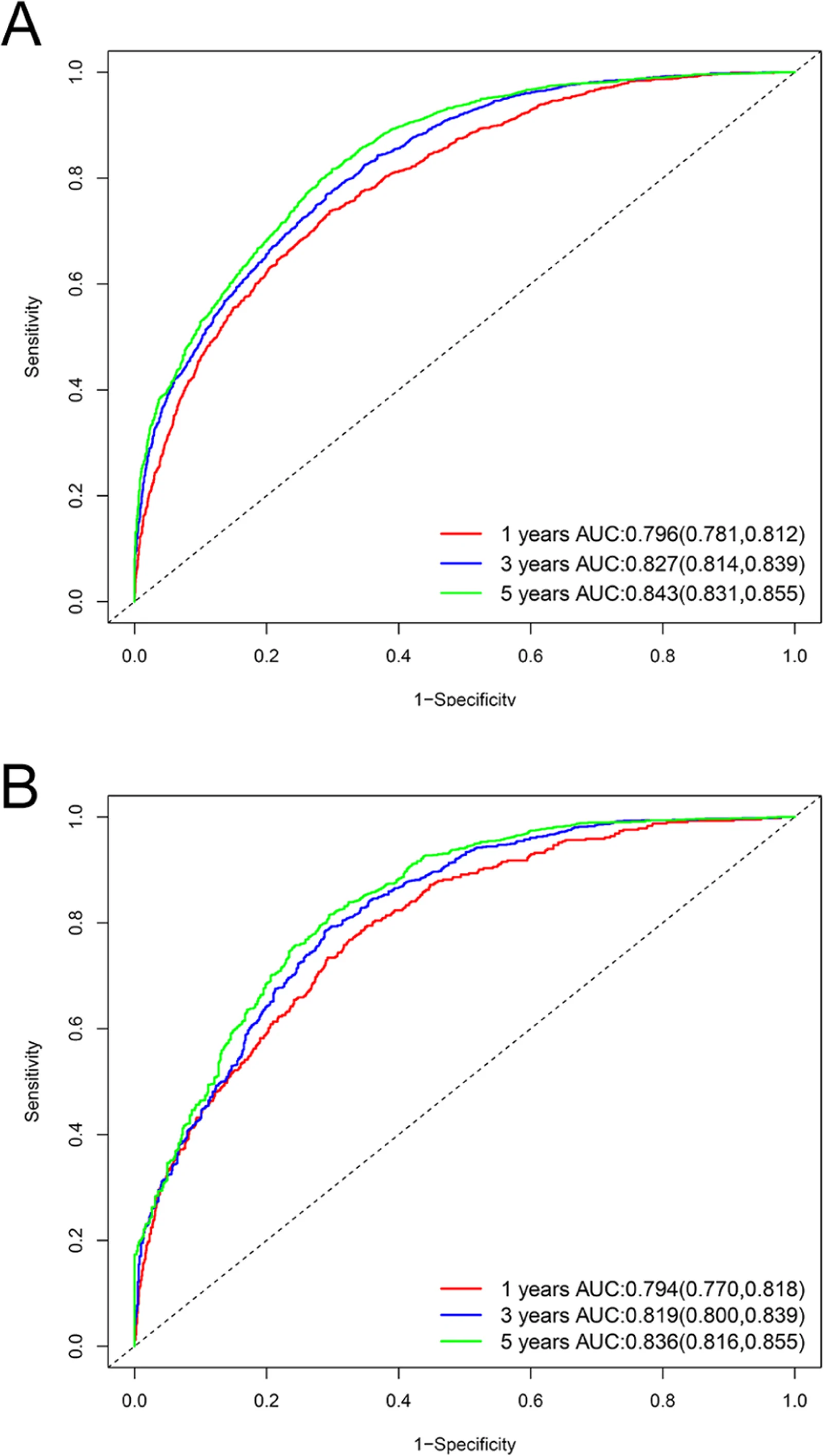
ROC curves of the nomogram for forecasting one-, three-, and five-year OS within the training cohort (A), and the validation cohort (B).

The C-indices for the training and validation cohorts were 0.740 (95% CI 0.722–0.758) and 0.740 (95% CI 0.712–0.768), respectively. This observation suggested the favorable discriminative capability of the model. The calibration curves for the training and validation cohorts demonstrated strong agreement between the one-, three-, and five-year predicted probabilities and observed outcomes of the nomogram (Fig. 2A and 2B), suggesting good accuracy of the nomogram. Within the training cohort, the AUCs of the nomogram for forecasting one-, three-, and five-year OS were 0.796 (95% CI 0.781–0.812), 0.827 (95% CI 0.814–0.839), and 0.843 (95% CI 0.831–0.855), respectively (Fig. 3A). Within the validation cohort, the AUCs for 1-, 3-, and 5-year OS were 0.794 (95% CI 0.770–0.818), 0.819 (95% CI 0.800–0.839), and 0.836 (95% CI 0.816–0.855), respectively (Fig. 3B). These results of AUC further confirmed the accuracy and discriminative performance of the nomogram.

### Univariate fine-gray analysis of cumulative incidence of CSD and death from other causes

**Fig. 4:**
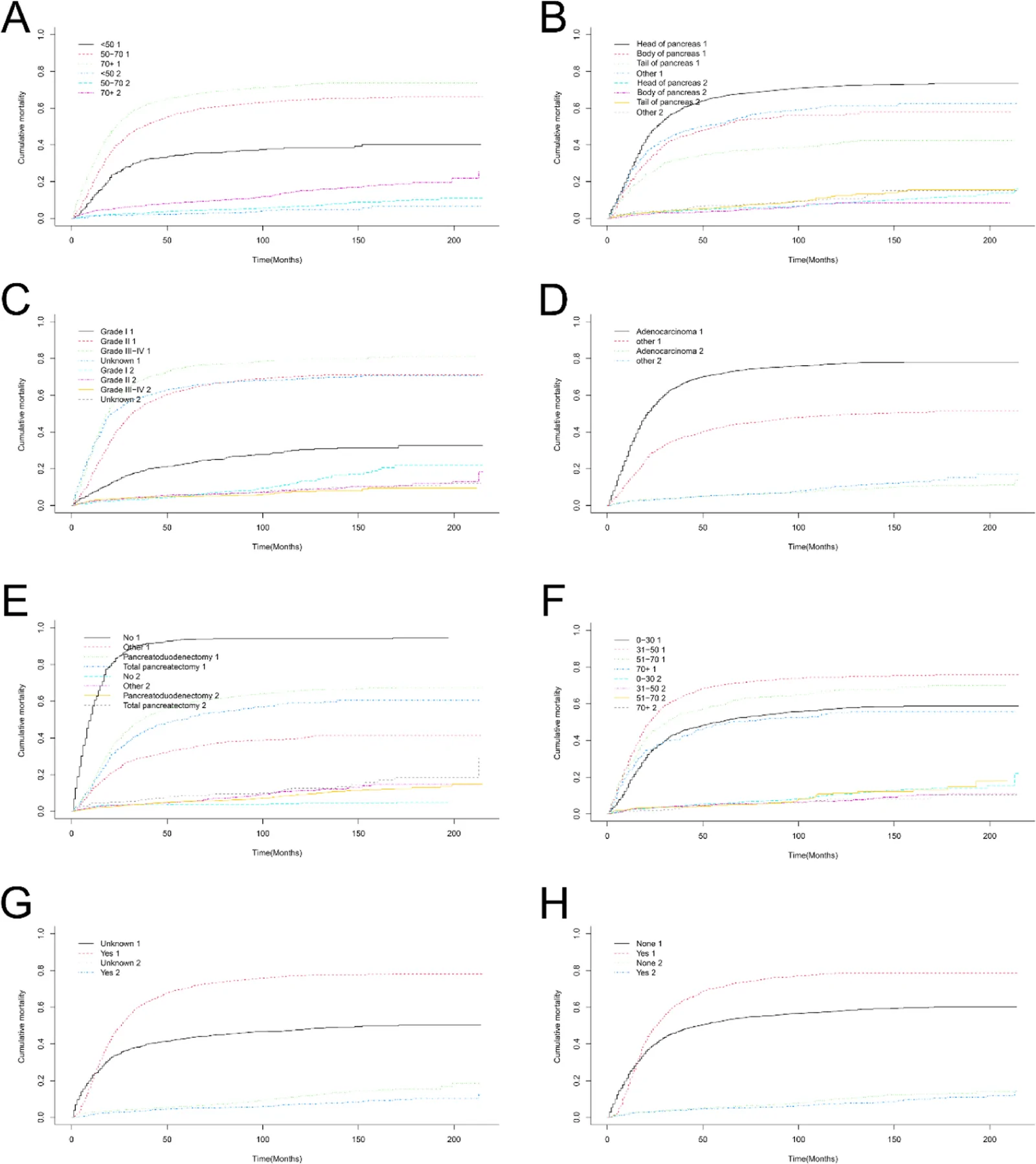
Cumulative incidence of cancer-specific death and death from other causes in lymph node-negative pancreatic cancer according to different factors: (A) Effect of age; (B) Effect of primary tumor site; (C) Effect of pathological grade; (D) Effect of histologic type; (E) Effect of surgery; (F) Effect of tumor size; (G) Effect of chemotherapy; (H) Effect of radiotherapy. Note: 1 Represents CSD, 2 Represents Death from Other Causes.

Univariate Fine-Gray analysis showed that, after stratifying by age, primary tumor site, pathological grade, histologic type, surgery, tumor size, chemotherapy, and radiotherapy, patients with lymph-node-negative pancreatic cancer consistently exhibited a significantly higher cumulative incidence of CSD than mortality from other causes (Fig. 4, cumulative incidence curves).

### Univariate CSS analysis: fine-gray

In the univariate assessment, numerous variables significantly influencing P were identified. These variables encompassed sex, age, marital status, primary tumor site, histologic type, pathological grade, AJCC TNM stage, surgery, tumor size, radiotherapy, and chemotherapy. Multivariate Fine-Gray analysis was performed to determine independent Subdistribution Hazard Factors (SHR) for CSS. The results confirmed that sex, age, marital status, primary tumor site, pathological grade, AJCC T stage, AJCC M stage, histologic type, surgery, and tumor size were independent prognostic factors for CSS (SHR values are shown in Table 3).

**Table 3:** Univariable and multivariable analyses of CSS in lymph node–negative pancreatic cancer.

**Factors**	**Define**	**Univariate**		**Multivariate**	
		**SHR (95% CI)**	***z*(p)**	**SHR (95% CI)**	***z*(p)**
Sex	Female	Ref.		Ref.	
	Male	1.13 (1.05‒1.22)	3.20 (0.001)	1.09 (1.00‒1.19)	2.03 (0.042)
Age	< 50	Ref.		Ref.	
	50‒70	2.03 (1.76‒2.35)	9.65 (<0.001)	1.52 (1.31‒1.76)	5.43 (<0.001)
	70+	2.69 (2.31‒3.12)	13.00 (<0.001)	1.96 (1.68‒2.30)	8.35 (<0.001)
Marital status	Married	Ref.		Ref.	
	Unmarried	0.86 (0.77‒0.97)	−2.44 (0.015)	1.02 (0.90‒1.16)	0.31 (0.760)
	Other	1.23 (1.13‒1.35)	4.49 (<0.001)	1.12 (1.01‒1.25)	2.23 (0.025)
Race	Black	Ref.			
	White	1.11 (0.97‒1.27)	1.55 (0.120)		
	Other	0.95 (0.79‒1.14)	−0.55 (0.580)		
Primary tumor site	250	Ref.		Ref.	
	251	0.67 (0.59‒0.76)	−5.99 (<0.001)	1.01 (0.87‒1.18)	0.10 (0.920)
	252	0.43 (0.38‒0.49)	−13.00 (<0.001)	0.80 (0.68‒0.94)	−2.68 (0.007)
	Other	0.75 (0.66‒0.85)	−4.67 (<0.001)	1.01 (0.88‒1.15)	0.09 (0.930)
Grade	Grade I	Ref.		Ref.	
	Grade II	3.33 (2.91‒3.82)	17.30 (<0.001)	2.21 (1.91‒2.56)	10.61 (<0.001)
	Grade III‒IV	4.79 (4.15‒5.54)	21.40 (<0.001)	2.94 (2.51‒3.45)	13.34 (<0.001)
	Unknown	3.80 (3.28‒4.41)	17.70 (<0.001)	1.71 (1.45‒2.01)	6.47 (<0.001)
AJCC_T	T1	Ref.		Ref.	
	T2	2.03 (1.72‒2.41)	8.18 (<0.001)	1.60 (1.34‒1.91)	5.22 (<0.001)
	T3	4.03 (3.47‒4.69)	18.10 (<0.001)	2.29 (1.94‒2.70)	9.73 (<0.001)
	T4	7.29 (6.08‒8.74)	21.54 (<0.001)	2.59 (2.10‒3.20)	8.89 (<0.001)
AJCC_M	M0	Ref.		Ref.	
	M1	2.71 (2.36‒3.10)	14.30 (<0.001)	1.87 (1.61‒2.17)	8.13 (<0.001)
Surgery	No Surgery	Ref.		Ref.	
	Pancreatoduodenectomy	0.29 (0.26‒0.32)	−22.70 (<0.001)	0.36 (0.32‒0.42)	−14.21 (<0.001)
	Total pancreatectomy	0.24 (0.21‒0.28)	−17.60 (<0.001)	0.33 (0.28‒0.40)	−11.93 (<0.001)
	Other	0.15 (0.13‒0.17)	−26.70 (<0.001)	0.30 (0.25‒0.36)	−12.87 (<0.001)
Radiotherapy	None	Ref.		Ref.	
	Yes	1.49 (1.38‒1.60)	10.20 (<0.001)	1.054 (0.96‒1.16)	1.08 (0.280)
Chemotherapy	Unknown	Ref.		Ref.	
	Yes	1.93 (1.77‒2.09)	15.40 (<0.001)	0.97 (0.87‒1.8)	−0.57 (0.570)
Size	0‒30	Ref.		Ref.	
	31‒50	1.68 (1.54‒1.82)	12.24 (<0.001)	1.23 (1.12‒1.35)	4.44 (<0.001)
	51‒70	1.36 (1.18‒1.56)	4.32 (<0.001)	1.10 (0.94‒1.29)	1.17 (0.240)
	70+	0.98 (0.83‒1.16)	−0.26 (0.790)	1.10 (0.91‒1.31)	0.99 (0.320)
Hist	Adenocarcinoma	Ref.		Ref.	
	Other	0.45 (0.41‒0.49)	−19.90 (<0.001)	0.75 (0.69‒0.83)	−6.14 (<0.001)

### Nomogram for CSS and its validation

**Fig. 5:**
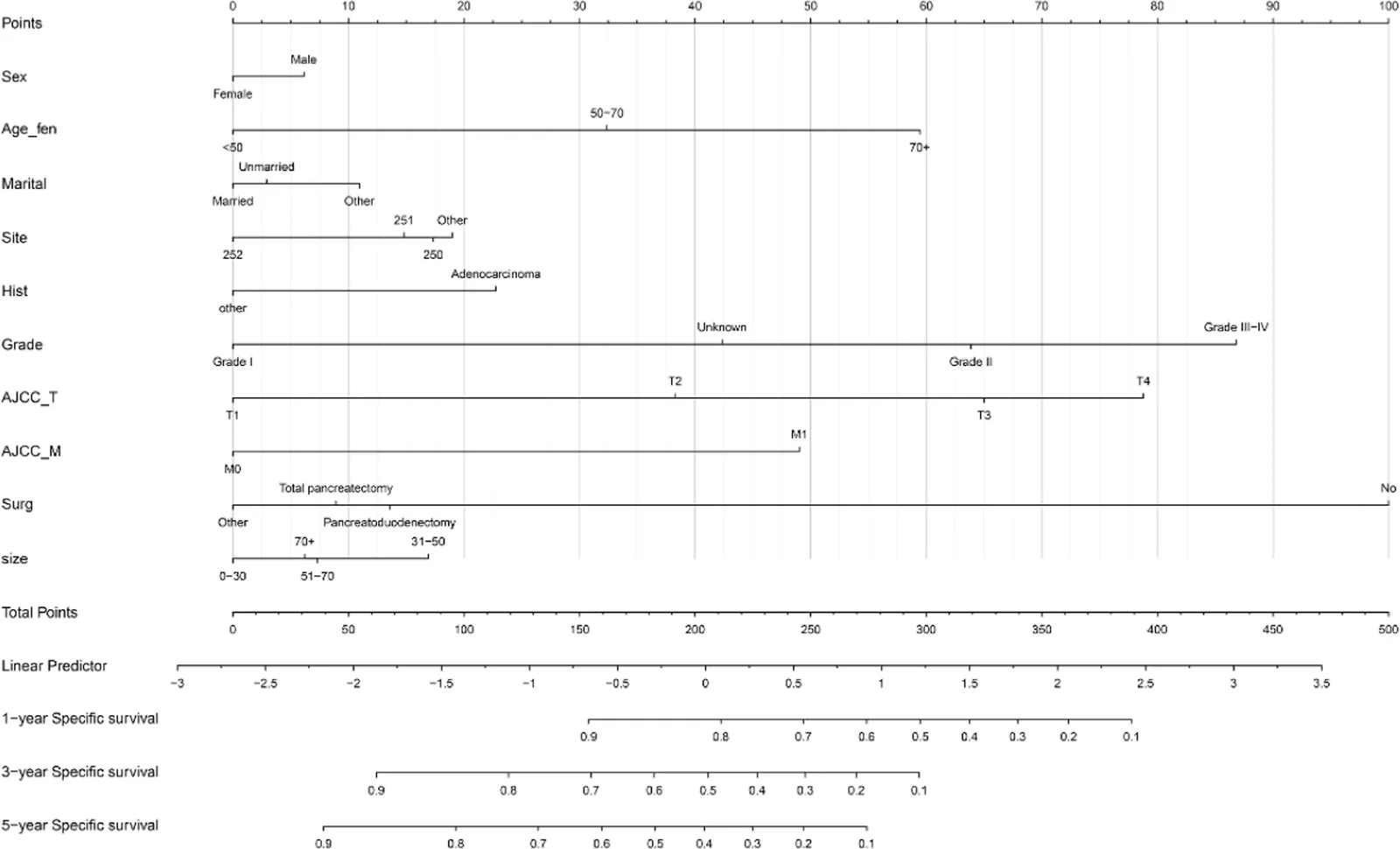
Nomogram for predicting one-, three-, and five-year CSS.

**Fig. 6:**
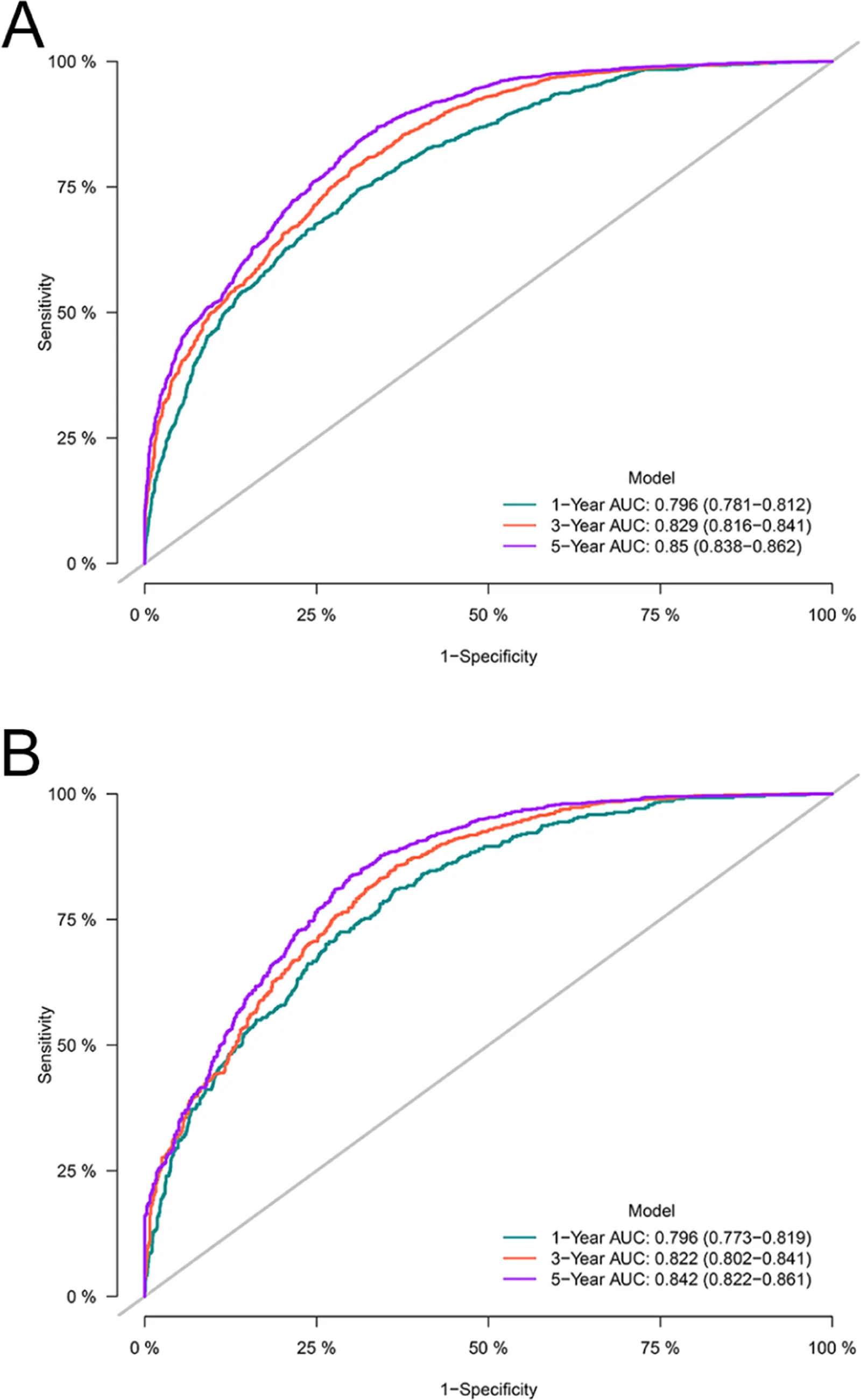
ROC curves of the nomogram for predicting CSS at varied time points within the training cohort (A), and the validation cohort (B).

**Fig. 7:**
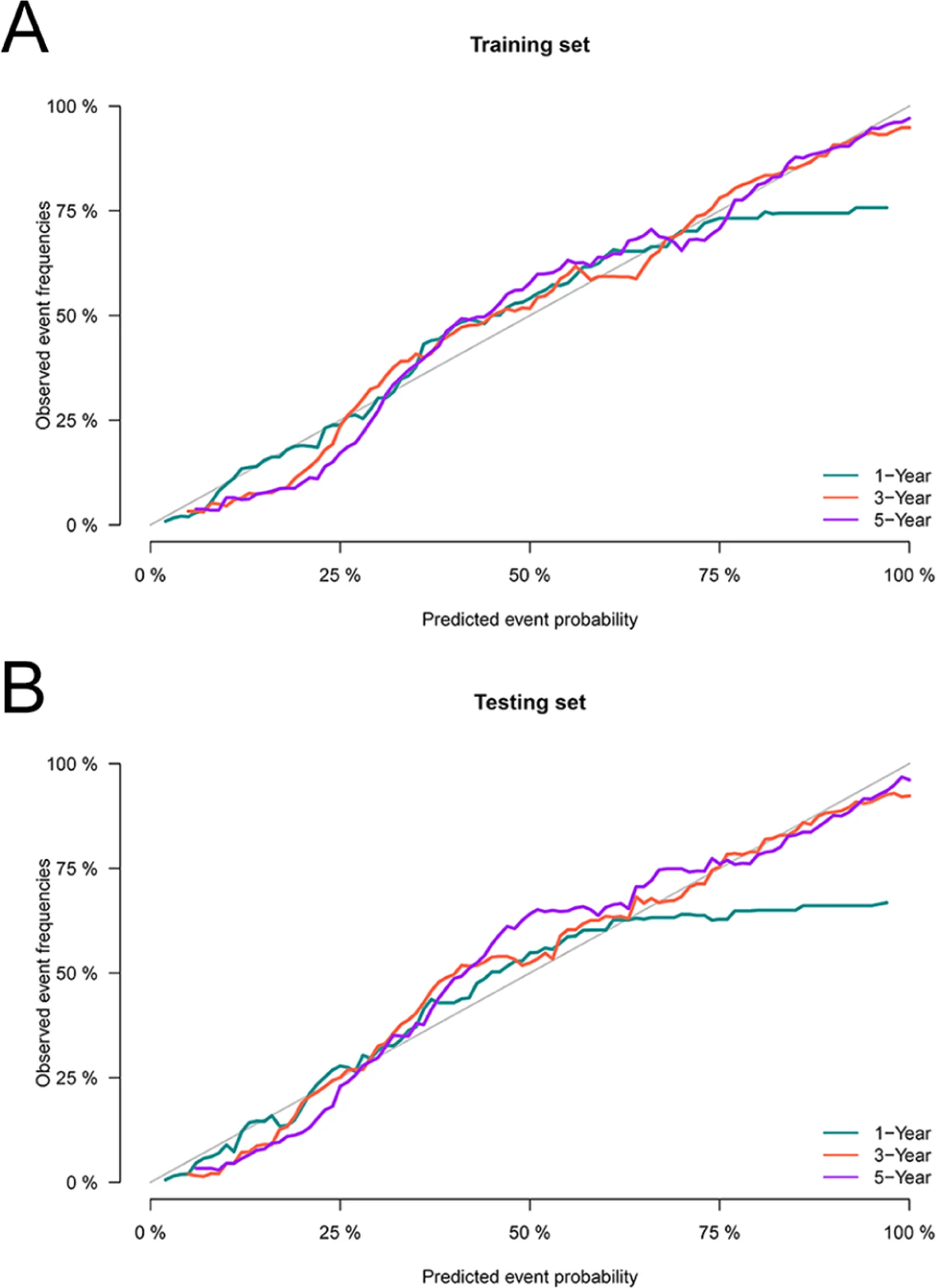
Calibration curves of the nomogram at varied time points within the training cohort (A), and the validation cohort (B).

Based upon the independent risk indicators identified above, a nomogram was developed to forecast CSS (Fig. 5). The C‑indices for the training cohort and the validation cohort were 0.737 (95% CI 0.719–0.756) and 0.736 (95% CI 0.708–0.764), respectively. Within the training cohort, the AUCs of the nomogram for one-, three-, and five-year CSS were 0.796 (95% CI 0.781–0.812), 0.829 (95% CI 0.816–0.841), and 0.850 (95% CI 0.838–0.862), respectively (Fig. 6A). Within the validation cohort, the AUCs of the nomogram for one-, three-, and five-year CSS were 0.796 (95% CI 0.773–0.819), 0.822 (95% CI 0.802–0.841), and 0.842 (95% CI 0.822–0.861), respectively (Fig. 6B). The results of AUC demonstrated the accuracy and discriminative capability of the nomogram. The calibration curves demonstrated close alignment with the ideal line across the training and validation cohorts. The predicted one-, three-, and five-year probabilities exhibited strong agreement with the observed outcomes (Figs. 7A and 7B). This indicated high predictive accuracy of the model. The DCA curve showed that, in comparison with the TNM staging system, the constructed nomogram exhibited superior clinical net benefit within both the training set and the validation set (Fig. S1 and S2).

### Estimation of cumulative mortality

According to conventional survival analysis, the one-, three-, and five-year cumulative CSD rates were 26%, 55%, and 63%, respectively. Conversely, the one-, three-, and five-year cumulative CSD rates estimated by the competing risks model were 24%, 51%, and 58%, respectively. At the same time points, the cumulative CSD rates determined by the competing risk model were persistently lower than those obtained from conventional survival analysis (Table S1).

### Sensitivity analysis

We further categorized the subjects based on whether lymph nodes were submitted for examination. Moreover, the robustness of the model was assessed exclusively within the non‑metastatic population. The predictive models constructed within each subgroup consistently demonstrated robust discriminatory ability (Fig. S3). Furthermore, sensitivity analysis restricted to M0 patients was performed. These findings revealed that the model exhibited comparable discriminatory power and calibration performance, thereby confirming the robustness of the nomogram (Fig. S4).

### Web-based calculator

Based upon the nomogram constructed above, a dynamic web-based application was developed using the shinyapp.io platform and is accessible through the following link: https://lanjingyu.shinyapps.io/DynNomapp/. This tool was internally validated using a randomly split subset from the same SEER database. External validation should be conducted to confirm the robustness of this tool across different populations.

## Discussion

Lymph node metastasis ranks among the important factors influencing the prognosis of pancreatic cancer. Though the prognosis of individuals with lymph node‑negative pancreatic cancer is significantly better relative to individuals with lymph node‑positive pancreatic cancer, the long‑term survival rate of individuals with lymph node‑negative pancreatic cancer remains unfavorable.^14^ Based on SEER database, a nomogram based on Cox and Fine-Gray competing risk model was constructed and validated to predict the OS and CSS of patients with lymph node-negative pancreatic cancer. This model demonstrated favorable discrimination, calibration, and predictive performance, thereby suggesting their strong clinical applicability. Furthermore, the Fine-Gray model achieved one-, three-, and five-year cumulative mortality rates of 24%, 51%, and 58%, respectively, which were lower than the one-, three-, and five-year cumulative mortality rates (26%, 55%, and 63%) obtained from traditional survival analysis. At 5-years, conventional survival analysis showed a cumulative CSD of 63%, whereas the competing‑risk model estimated 58%. Although the absolute difference is modest, this overestimation by conventional methods is clinically meaningful: it may lead to inflated risk perception, more aggressive adjuvant treatment, or intensified surveillance. The competing‑risk model provides a more realistic and conservative estimate of CSD.

Through univariable and multivariable analyses, this research observed that sex and age are independent risk indicators affecting patients with lymph node-negative pancreatic cancer. Older patients (> 70-years) have a poorer prognosis, whereas younger patients have a better prognosis. This might be linked to age‑associated decline in physiological function and impairment of immune function.^15^ We also observed a lower CSD rate among female patients. Nevertheless, current research shows no clear evidence that sex is a core indicator influencing the prognosis of pancreatic cancer. Numerous studies have demonstrated[16,17] that the location of pancreatic tumors is a pivotal factor influencing the prognosis of patients. However, associations between varied locations and prognosis remains controversial. Zhang HQ et al.^16^ have found that individuals with pancreatic head cancer demonstrated superior OS relative to those with body or tail cancers, regardless of whether metastases were multiple or confined solely to the liver or lungs. This may be due to pancreatic body‑tail cancers enhancing their malignancy and immune evasion capacity by activating pro‑invasive gene programs and inducing immunosuppression, thereby leading to more aggressive biological behavior. This survival advantage reflects the ability of pancreatic body or tail cancers to activate pro-invasive gene programs and induce immunosuppression, thereby enhancing their malignant potential and immune-evasion capacity and leading to more aggressive biological behavior. Winer LK et al.^17^ have reported that pancreatic head cancer exhibited higher rates of lymph node positivity, more advanced tumor staging, and more frequent advanced pathological disease relative to pancreatic body or tail cancers. Our findings further reveal that among individuals with lymph node-negative pancreatic cancer, those with body or tail cancers showed better survival outcomes. This phenomenon might be linked to the propensity of pancreatic head cancers toward distant metastasis and recurrence.

This research indicates that surgery was the most important factor affecting OS and CSS among individuals with lymph node‑negative pancreatic cancer. Among all patients, individuals who received surgical intervention demonstrated markedly improved survival outcomes relative to non-surgical cohorts. One study has exhibited^18^ that patients with early-stage, resectable stage Ia pancreatic cancer have a five-year survival rate of approximately 84%. Notably, continued refinements in minimally invasive techniques are expanding surgical indications and offering curative opportunities to an increasing number of patients. Moreover, poorer tumor pathological grade (especially poorly differentiated or undifferentiated tumors) reflects the highly aggressive biological behavior of pancreatic cancer and contributes to unfavorable prognosis. An analysis based upon a large‑scale cohort (n = 57,804) has indicated^19^ that the median OS of pancreatic ductal adenocarcinoma was 20.2-months, significantly shorter than other pathological subtypes such as squamous cell carcinoma, mixed acinar-neuroendocrine carcinoma, and mucinous cystic neoplasms with associated invasive carcinoma. This finding aligns with our results and confirms that ductal adenocarcinoma is a potential adverse prognostic factor. This research also reveals that chemotherapy improved OS in these populations (multivariable HR = 0.90; 95% CI 0.82‒0.98). FOLFIRINOX or gemcitabine‑based combination chemotherapy has become the front-line treatment for pancreatic cancer,^20^^,^^21^ and neoadjuvant chemotherapy has further increased the resectability of tumors.^22^ With the favorable outcomes achieved by targeted therapy and immunotherapy in various solid tumors, more therapeutic options have become available for individuals with pancreatic cancer. In the KEYNOTE‑158 clinical trial, pembrolizumab achieved an objective response rate of 34.3% among individuals with pancreatic cancer. The median duration of response could not be determined, demonstrating durable antitumor activity.^23^ In the POLO clinical trial, among individuals with metastatic pancreatic cancer harboring germline BRCA1/2 mutations, maintenance therapy with olaparib significantly prolonged median progression‑free survival from 3.8-months to 7.4-months relative to placebo and diminished the risks of disease progression or death by 47%.^24^

During the construction of the prognostic model for individuals with lymph node-negative pancreatic cancer based on the SEER database, accurate assessment of the risk of CSD is the core challenge. An important methodological contribution of this study lies in comparing the differences between the traditional Cox proportional hazards model and the Fine-Gray competing risk model in identifying risk indicators. In traditional Cox models which analyze a single endpoint, competing events (such as cardiovascular or other non-cancer deaths) are typically treated as censored. However, this practice violates the fundamental assumptions of the model and results in biased estimates of the cumulative incidence of CSD.^25^ The oversight of competing risks, which can lead to erroneous conclusions, is not uncommon in oncological prognostic research.^26^

The Fine-Gray model directly models the cumulative incidence function for a specific endpoint and thus provides more reliable risk assessments amidst competing risks.^27^ Our findings corroborate this advantage. For instance, when the age is evaluated, the Cox model may overestimate its effect on CSD owing to inherent limitations. Nevertheless, the Fine-Gray model can more clearly disentangle the confounding effect of age as a strong risk factor for non-cancer mortality. This observation is consistent with the advantages demonstrated by the Fine-Gray model in competing risk modeling for other malignancies, such as cervical cancer and colorectal cancer.^28^^,^^29^ As far as we know, despite the increasingly widespread application of competing risks models, a CSD model focused specifically on lymph node-negative pancreatic cancer remains lacking. Therefore, this study provides a clinical tool for risk stratification and offers a more rigorous methodological reference for future investigations.

This research is pioneering in constructing a prognostic prediction model incorporating competing risks specifically for lymph node-negative pancreatic cancer based upon a large-scale population-base database. This is the core strength of our analysis. Relative to the conventional TNM staging system, this model translates the results of multivariable analysis into an intuitive clinical nomogram, enabling quantitative assessment of individualized risks and facilitating the identification of high-risk lymph node-negative cases who cannot be distinguished by conventional staging.

This research has several limitations. First, treatment variables (surgical resection and chemotherapy) in the SEER database are observational and inherently subject to confounding by indication. Patients with better performance status, fewer comorbidities, and more favorable tumor biology are systematically more likely to receive aggressive therapy, which may inflate the apparent prognostic value of these variables. Consequently, the model coefficients for treatment should not be interpreted as causal estimates or as direct evidence supporting one therapeutic approach over another. Rather, the nomogram predicts real-world outcomes under standard clinical management. It should be applied as a prognostic tool after definitive therapeutic strategies have been determined. Second, the SEER database lacks critical clinicopathological and laboratory variables significantly associated with prognosis, including resection margin status, perineural invasion, vascular invasion, CA19‑9 levels, performance status, and comorbidity indices. In addition, molecular markers such as KRAS and BRCA mutations are not available in the SEER database, which may further restrict the predictive accuracy and clinical applicability of the models. Furthermore, the database lacks detailed information on chemotherapy regimens, dosing, duration, and treatment completion rates, which constrains both the precision of analysis and the forecasting performance of the model. Third, as a retrospective study, residual confounding cannot be completely ruled out despite comprehensive efforts to control for bias. Finally, the model has undergone only internal validation through random splitting of the same SEER dataset, which may introduce optimism bias and does not represent true external validation. Its generalizability requires independent external validation using data from different populations and institutional cohorts. Temporal validation (e.g., early versus late diagnosis periods) could further strengthen the robustness of the model and is recommended for future studies.

## Conclusion

In this study, we developed and internally validated a risk-based competing nomogram designed to individually predict the OS and CSS rates of patients with lymph node-negative pancreatic cancer, utilizing data from the SEER database. The model exhibits favorable performance in discrimination and calibration. Therefore, it can serve as a practical tool for prognostic evaluation and risk stratification in clinical practice. By identifying lymph node-negative patients with an inherently high risk of CSD, this nomogram facilitates personalized follow-up planning and clinical assessment. Prospective studies incorporating more comprehensive clinicopathological and molecular data should be conducted in the future to externally validate and further refine the model.

## Ethics approval and consent to participate

This study utilized publicly available, de-identified data from the SEER database. Therefore, ethical review approval and the requirement for informed consent were waived. The research was conducted in accordance with the principles of the Declaration of Helsinki and complied with the SEER data-use agreements.

## Consent for publication

Not applicable.

## Data availability statement

The datasets analyzed in this study are available from the SEER database of the National Cancer Institute, USA: https://seer.cancer.gov/seerstat/.

## Authors’ contributions

All authors contributed to the study conception and design. Writing-original draft: Zheyuan Wang; Writing-review and editing: Zheyuan Wang, Lu Zhang; Conceptualization: Zheyuan Wang; Methodology: Zengyou Li; Formal analysis and investigation: Lu Zhang; Funding acquisition: Zheyuan Wang; Resources: Huihan Zhang; Supervision: Zheyuan Wang, and all authors commented on previous versions of the manuscript. All authors read and approved the final manuscript.

## Funding

This study was funded by the 10.13039/501100004775Natural Science Foundation of Gansu Province (n° 23JRRA1621) and Gansu Province Drug Regulatory Scientific Research Project (n° 2024GSMPA032).

## Conflicts of interest

The authors declare no conflicts of interest.

## Appendix Supplementary materials

Image, application 1
