# Supplementary material for: Web-based dynamic nomogram for forecasting overall survival and cancer-specific survival among individuals with lymph node-negative pancreatic cancer: based upon the SEER database
Source: Clinics (Sao Paulo). 2026 Sep 7;81:101100. doi: 10.1016/j.clinsp.2026.101100 (PMC13579279; doi:10.1016/j.clinsp.2026.101100)
Supplement: Supplementary file 1 [file mmc1.docx]

CLINICS-D-25-02220_Supplementary Material

**Table S1** Cumulative mortality rates at varied time points.

| **Time (Year)** | **Conventional survival analysis** | **Competing risk model** | |
| --- | --- | --- | --- |
|  |  | **Cancer-specific death** | **Death from other causes** |
| 12 | 0.26 | 0.24 | 0.03 |
| 24 | 0.46 | 0.42 | 0.03 |
| 36 | 0.55 | 0.51 | 0.04 |
| 48 | 0.60 | 0.55 | 0.05 |
| 60 | 0.63 | 0.58 | 0.05 |
| 72 | 0.66 | 0.60 | 0.06 |
| 84 | 0.67 | 0.61 | 0.06 |
| 96 | 0.69 | 0.62 | 0.07 |
| 108 | 0.69 | 0.63 | 0.08 |
| 120 | 0.70 | 0.64 | 0.09 |
| 132 | 0.71 | 0.65 | 0.10 |

**Figure S1** DCA curves for OS: (A) One-year training cohort; (B) Three-year training cohort; (C) Five-year training cohort; (D) One-year validation cohort; (E) Three-year validation cohort; (F) Five-year validation cohort.

**Figure S2** DCA curves for CSS: (A) One-year training cohort; (B) Three-year training cohort; (C) Five-year training cohort; (D) One-year validation cohort; (E) Three-year validation cohort; (F) Five-year validation cohort.

**Figure S3** ROC curves of the nomogram for lymph node subgroup analysis: (A) ≥12 lymph nodes for OS; (B) ≥12 lymph nodes for CSS; (C) <12 lymph nodes for OS; (D) <12 lymph nodes for CSS.

**Figure S4** ROC curves of the nomogram for M0 patients: (A) OS; (B) CSS.
